# Effect of a resistance and balance exercise programme for women with osteoporosis and vertebral fracture: study protocol for a randomized controlled trial

**DOI:** 10.1186/s12891-018-2021-y

**Published:** 2018-04-03

**Authors:** Brita Stanghelle, Hege Bentzen, Lora Giangregorio, Are Hugo Pripp, Astrid Bergland

**Affiliations:** 1 Faculty of Health Sciences, Department of Physiotherapy, Oslo and Akershus University College of Applied Sciences, PO box 4, St. Olavs plass, 0130 Oslo, Norway; 2 Department of Physiotherapy, Oslo and Akershus University College of Applied Sciences, Oslo, Norway; 3 Department of Kinesiology, University of Waterloo, Waterloo, Canada; 4 Faculty of Health Sciences, Oslo and Akershus University College of Applied Sciences, Oslo, Norway; 5 Faculty of Health Sciences, Leader of the research group Age, Health and Welfare and the PhD- program in Health Sciences, Oslo and Akershus University College of Applied Sciences, Oslo, Norway

**Keywords:** Osteoporosis, Vertebral fracture, Exercise, Physical function, Health related quality of life

## Abstract

**Background:**

Osteoporotic vertebral fractures are common, and are associated with reduced functioning and health related quality of life. The primary aims of this randomized controlled trial are to examine the immediate and long-term effects of a 12-weeks supervised group exercise programme on habitual walking speed in older women with osteoporosis and a history of vertebral fracture. The secondary aims are to examine the immediate and long-term effects of the exercise program on physical fitness, fear of falling and quality of life.

**Methods:**

The study is a single-blinded randomized controlled trial. Women aged 65 years or older with osteoporosis and a history of vertebral fracture are included. The intervention group receives a 12-week multicomponent exercise programme, including resistance training combined with balance training. The control group receives usual care. Adherence to the programme will be of importance for the internal validity of the study. Participants in the exercise group who don’t attend will be followed up with motivational phone calls. The primary outcome is habitual walking speed over 10 m. Secondary outcomes are health related quality of life (Qualeffo-41, SF-36), physical activity (I-PAQ), Patient Specific Functional Scale, Fear of falling (FES-1) and physical fitness (Senior Fitness test, Functional reach test, 4 square step test, grip strength). Sample size, based on the primary outcome, is 150 participants randomized into the two arms on a 1:1 allocation, including an estimated 20% drop out. Descriptive data will be reported as mean (standard deviation), median (range) or count (percent) as appropriate. The data will be analysed following the intention-to-treat principle. Between group differences in primary and secondary outcomes at 3 months follow-up will be assessed using linear regression models with respective outcome at baseline as covariate and the randomised group as factor.

**Discussion:**

This trial will generate new knowledge on the effects of a multicomponent exercise programme among women over 65 years with osteoporosis and a history of vertebral fracture, knowledge that is of importance for clinicians, health managers and policy makers.

**Trial registration:**

ClincialTrials.gov Identifier: NCT02781974. Registered 18.05.16. Retrospectively registered.

## Background

Osteoporosis is a worldwide health problem. Approximately 30% of all postmenopausal women in Europe and the United States have osteoporosis. More than 40% of these women will sustain one or more fragility fractures in their remaining course of life [1] Osteoporosis is usually defined as an overall bone mineral density (BMD) that is less than or equal to 2.5 standard deviations below the mean BMD for young, female adults [2]. The prevalence of osteoporosis is more frequent among women than men [2]. Osteoporosis is a systemic skeletal disease characterised by low bone mass and deterioration of bone tissue leading to bone fragility and an increased risk of fractures [2].

An osteoporotic fracture, also called a fragility fracture, is a fracture that occurs with minimal trauma [3]. Vertebral fractures, the most common type of osteoporotic fractures, are associated with height loss, kyphosis, back pain and reduced balance, mobility and activity of daily life (ADL), as well as reduced physical activity [4, 5]. Women with osteoporotic vertebral fractures have an increased risk of subsequent vertebral fractures [6] and experience reduced health related quality of life (HRQOL) compared to those who have not experienced fractures [2, 7, 8]. Several studies have shown that people with vertebral fractures experience reduced HRQOL, both in short and long term perspective [9–11]. Further, women with fragility fractures are more likely to experience fear of falling, anxiety, depression and loss of social roles [12–15].

Good physical fitness can contribute to maintenance of functional independence in older people [16]. According to Rikli and Jones [17] physical fitness is defined as the capacity to perform activities of daily living safely and independently without fatigue. The concept is multidimensional and includes muscle strength, aerobic endurance, flexibility, body composition, dynamic balance and agility/mobility [17]. Recent recommendations specify that older adults with osteoporosis or osteoporotic vertebral fractures should engage in a multicomponent exercise programme that includes resistance training combined with balance training [18, 19]. The mode of exercise, dosage and effects on different outcomes for this group has not yet been well established by research [3, 20]. Safety considerations are necessary when it comes to exercise for individuals with vertebral fractures since adverse events have happened in previous randomized trials [3, 18, 19]. Examples include modifying or avoiding rapid, repetitive, weighted and sustained or end-range flexion or twisting of the spine [18, 21]. Although individual trials of exercise in individuals with vertebral fractures did report benefits for some pain, physical function and quality of life outcomes, the quality of evidence is low. A recent Cochrane review concluded that there is insufficient evidence to determine whether exercise interventions reduce fear of falling beyond the end of the intervention, and their effect on other outcomes from designed randomised trials is required [22]. However, Olsen and Bergland [23] concluded that a group-based exercise programme and an educational session had a positive and durable effect on fear of falling in community-dwelling elderly women with osteoporosis and a history of vertebral fracture.

The main focus of this project is to develop evidence-based knowledge regarding exercise as an intervention for improving physical fitness and HRQOL in older women with osteoporosis and a history of vertebral fracture. Finding safe and feasible interventions to improve physical fitness and increase HRQOL in this group will have great potential benefits. This study builds on a previous study conducted by Bergland et al. [24]. However, the intervention in this study will to a greater degree conform to recent exercise recommendations regarding dosage and difficulty level [18]. In this trial we have left out the education component to be able to explore solely the effect of exercise, and we aim to include a larger sample.

### Aims

#### Primary aim

To assess the effects of a 12-weeks supervised strengthening and balance programme on habitual walking speed among older women with osteoporosis and a history of vertebral fracture.

#### Secondary aims

To assess the effects of the same programme on physical fitness, fear of falling and health related quality of life among women with osteoporosis and a history of vertebral fracture.

### Hypotheses

An intervention consisting of a 12- weeks supervised group exercise programme will improve habitual walking speed as well as physical fitness, fear of falling and quality of life among older women with osteoporosis and a history of vertebral fracture.

## Methods/design

### Study design

**Fig. 1:**
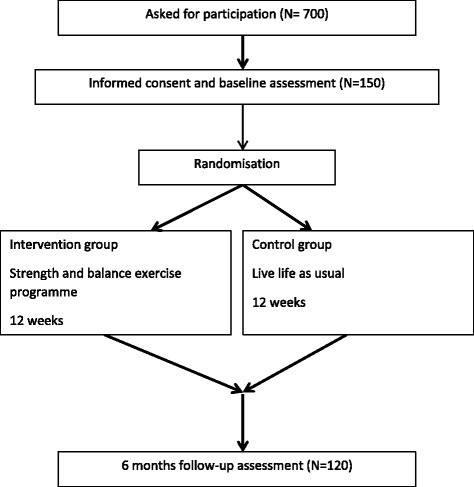
Flow of participants in the study

This is a parallel-group, single blinded randomized controlled trial. The participants will be randomly assigned in a 1:1 ratio to the intervention group and the control group after baseline assessment. A computer-generated, permuted block randomization scheme is used to allocate the participant, block sizes varying from 4 to 8. The allocation of the participants is administered by an individual not involved in any testing or contact with the participants, and the allocation sequence is kept on that individual’s computer. Following randomisation, the participant receives information by telephone on which group they are allocated to. See flow chart in Fig. 1.

### Study setting

The present study will be undertaken at facilities in Oslo and Akershus University College of Applied Sciences, and at two different community based facilities in the area around Oslo, Norway.

### Recruitment and study population

Participants will be recruited from a specialty outpatient clinic for osteoporosis in Oslo, and from two outpatient clinics at hospitals in and around Oslo.

### Exclusion/inclusion

We will include women at the age of 65 or over, living at home and able to walk independently with or without walking aid. Further, to be found eligible, the women must have a t-score − 2.5 SD or less verified by Dual X-ray Absorptometry (DXA) scan and at least one vertebral fracture classified grade 1, 2 or 3 [6] verified by DXA or X-ray. Patients will be excluded if they are not able to speak and understand Norwegian. Individuals who have self-reported severe diseases or other health conditions like severe lung- or heart diseases that make it unsafe to exercise will be excluded. In cases of uncertainty the participants will be asked to consult their physician to make sure that it is safe for them to exercise.

### Intervention group

The intervention will be informed by recent exercise recommendations for people with osteoporosis and vertebral fracture [18], which recommend progressive resistance training for all major muscle groups in combination with balance training. The expert panel recommends that older people with osteoporosis and vertebral fracture do not engage in aerobic exercise to the exclusion of resistance and balance training [18]. The recommendations for exercise prescription for people with osteoporosis and vertebral fracture are based on the GRADE process [3] to evaluate the quality of existing evidence and generate recommendations for exercise prescription [18]. The programme should be performed at a minimum of twice a week. Alongside with the mentioned recommendations [18], the exercise program of this study is also based on the guidelines from the position stand on Exercise and Physical Activity for Older Adults from the American College of Sports Medicine [25], recommendations for fall prevention exercises [26] and guidelines for treatment in postmenopausal and senile osteoporosis [27].

The intervention is designed as a group-based circuit exercise programme of eight different exercises. Emphasis is put on resistance and balance exercises with some aerobic components as well. The majority of the exercises will be carried out in a weight bearing position, with or without support. Besides different exercises to improve leg strength and balance, exercises for upper limb strength, upper and lower back strength and posture are also integrated in the programme. The intervention will be led by an experienced physiotherapist. The participants with increased risk of falls, restrictions in movement or weight-bearing during training and activity will be focused upon to avoid injuries. The physiotherapists will be trained in instructing the resistance and balance exercise programme and to make sure the participants have support objects nearby when necessary.

The programme will last for 1 h and will be carried out twice a week for 12 weeks. The physiotherapist supervising the exercise session, will also be responsible for individual tailoring and suitable dosage and progression of the exercises. The exercise session starts with 10 min warm up consisting of stretching, breathing exercises, flexibility and dynamic balance carried out with background music. The warm up is followed by two rounds of stationary circuit sessions with strength- and balance exercises. The participants will work 1.5 min at each station, with a short break of 30 s to rest and move to the next station. The exercise session ends with 10 min cool down and stretching. The participants are encouraged to report any serious adverse events caused by the exercise like muscle soreness, joint tenderness and increased level of pain.

When designing the exercise programme all the chosen exercises were evaluated according to safety cautions for people with osteoporosis and vertebral fractures [3, 18, 21]. Exercises that includes flexion and rotation of the spine are not considered safe and therefore not included in the programme. We also had in mind that the participants should be able to support themselves on a chair or a bar when performing exercises that challenged the balance if needed. Correct techniques in the different exercises is also a priority in the programme, and for the two first weeks of the programme this is emphasized for the participants by thoroughly instructions of the physiotherapists. The exercise group will have no more than 8–10 participants at the time, making sure that the physiotherapist can supervise and instruct the participants in a safe way. After a few weeks of adaption, the participants are encouraged to intensify their exercise and work until volitional fatigue without jeopardizing safety so that they perceive it as somewhat hard. This corresponds with an intensity level of 13–14 on the Borg Rating of Perceived Exertion Scale [28]. Weight belts, elastic bands, manual weights and frequency will be used for the progression of the strength exercises. Advancing to more challenging positions will be used to progress in balance exercises [29]. See Table 1 for a detailed description of the exercises.

**Table 1:** Resistance and balance programme, description of exercises

Exercise	Therapeutic goal	Level	Description	Progression or modify
Squats	Leg strength	1	Raise from a chair (sit to stand)	Tempo, weightbelt
		2	Deep squats (lower than 90 degrees kneeflexion)	Weightbelt, Degrees of kneeflexion
		3	Lounges	Weightbelt
Step up	Leg strength, dynamic balance	1	Go up and down from a step, change legs	Height of steps, tempo, Weightbelt. support
		2	Step up and one leg stance, go down	Tempo, height of steps
Sideways step up	Hip stability, dynamic balance, leg strength	1	Step up sideways on a step	Height of the step, tempo, weightbelt
Upright row	Posture, upper back strengthening	1	Row exercise with elastic bands	Three different resistance levels on the elastic bands
Balance pad	Balance, static and dynamic	1	Standing on balance pad, semitandem	Open/closed eyes, turning head from one side to the other
		2	Standing on balance pad, tandem	
		3	One leg standing	
Chest press	Chest and arm strengthening	1	Chest press against the wall	
		2	Chest press against a bench	
		3	Chest press resting on the knees on the floor	
Hip raise	Lower back strengthening	1	Lying on back with knees bent, raise hip	
		2	One leg hip raise	
Diagonal lift		1	Standing on all four, lift left arm and right leg, alternate	Choose either hip raise or diagonal lift
Biceps curl	Upper arm strengthening	1	Seated armcurls with dumbbells	Increase load of dumbbells (1, 2, 3, 4 or 5 kg)

### Control group

Participants allocated to the control group receive treatment as usual. They are asked to maintain their current physical activity level and continue life as usual. They will be contacted for their follow up tests at 3 months and 6 months. After the 6-months test, participants in the control group are offered to participate in the same strength and balance exercise programme as the participants of the intervention group.

### Time plan of the study

Recruitment started January 2016 and is expected to be completed by spring 2018. The intervention will continue 12 weeks after the last inclusion. Data collection will last for 6 months after recruitment is completed. Thereafter, we will write up and publish peer-reviewed articles.

### Outcome measures

Assessments will be performed at baseline, and at 3 and 6 months. The participants are randomised after the baseline assessment by a person not involved in the recruitment, assessment or intervention of the study. Assessors (certified physiotherapists) are blinded to participants’ group allocation for all outcomes and the participants are instructed not to reveal group allocation to the assessors during the study period. The assessors are taking part in a training programme to ensure consistency in how the tests are performed and to ensure that the protocol is standardized. The time window between baseline assessment and start of intervention is aimed to be within two week(s) and the same time window for assessment due at 3 and 6 months.

### Adverse events

Adverse events such as falls, pain, fracture, and joint pain will be recorded by the instructor of the group during the exercise sessions. The participants will be instructed to report any adverse events outside the exercise session to the instructor of the group.

### Demographic variables and descriptive

We will record the following variables: age, living alone (yes/no), BMI, smoking (yes/no), are you afraid of falling (yes/no), have you fallen the last year (yes/no), injuries caused by falls the last year, number and name of medications, taking analgesics (yes/no), pain level last week by score from 0 to 10 on a Numeric Rating Scale [30, 31] and comorbidities.

### Primary outcome

Physical function, measured by 10 m habitual walking speed, is the primary outcome of this study. Walking speed is considered as a robust measure and is a valid, reliable and sensitive measure for assessing functional status and overall health in a wide range of populations [32, 33]. Walking speed is indicative of an individual’s functional capacity and general health status, but also predictive of a range of outcomes including rehabilitation response, frailty and mobility disability. There is also an observed association between slow selected walking speed and lower quality of life [32, 34].

There is no standardized protocol available for measuring walking speed, and a variety of procedures exist that differ in regards to distance [32, 35]. In this study habitual walking speed of a straight path of 10 m will be measured from a static position, using a stopwatch as timing instrument. No acceleration or deceleration phase will be used. The stopwatch is started as the participant crosses the start line with her first foot, and stopped when crossing the finishing line with the first foot [35]. The participant will be instructed to walk at a comfortable pace, in the speed she normally chooses when walking from one point to another. A walking aid can be used when necessary, and the test will be performed with the same walking aid at the post tests if needed.

### Secondary outcomes

Secondary outcomes are physical fitness, HRQOL, Falls-efficacy Scale International (FES-1) and International Physical Activity Questionnaire short form (IPAQ –SF).

#### Physical fitness

Alongside habitual walking speed as a measure of physical fitness, we apply tests like functional reach (FR), the four square step test (FSST), grip strength and Senior Fitness Test (SFT).

FR is a reliable and valid measure of proactive balance [36], and is a sensitive measure strongly connected to physical frailty [37]. FR is a measure of the maximal distance one can reach forward from a standing static position. The test will be performed three times, after one test trial to make sure that the participant understands the instructions. A mean of three recorded attempts will be reported.

FSST is a balance test for dynamic standing balance [38]. Four sticks or canes are resting on the floor forming four squares. The participant is instructed to step as fast as she can from square to square, with both feet resting in one square before she can move to the next. The FSST will be performed starting in square 1 then moving to 2,3,4,1,4,3,2,1. This test requires that the participant steps forward, backward and sideways, both to the left and right. A stopwatch is used to measure time it takes to complete, starting when the first foot touches square 2 and stops when the last foot has landed in square 1. The participant will first be shown the test, before one trial attempt is performed. The best of two successful attempts will be recorded as the score. Tested on a group of community dwelling elderly adults, the FSST is shown to be valid and reliable test, with a sensitivity of 85% and a specificity of 88–100% [38].

Grip strength is measured with a hydraulic handheld dynamometer, using the second position of the handle position for all participants. The handgrip test is a simple and reliable [39] method of measuring muscle strength [40]. Grip strength measurement has shown to have predictive validity, and falls, disability, low health related quality of life have been associated with low values of grip strength [40]. The test will be performed with the subject sitting at a chair, holding the instrument with the shoulder in a neutral position, elbow flexed to 90 degrees. The participant will perform one submaximal test of each hand, before performing three maximal tests of each side. The dominant hand will be tested first. The highest score of each hand is recorded.

SFT is a valid and reliable test for physical fitness [41] designed to assess underlying physical components associated with mobility in older people, such as muscle strength, aerobic endurance, flexibility, body composition (assessed as BMI in kg/m2) and agility/dynamic balance [42]. The test consists of: number of Chair Stands in 30 s, number of Arm Curls in 30 s, Chair-Sit-and-Reach-Test (CSRT) (cm), Back Scratch Test (cm), 2.45 m Up-and-Go test (seconds) and 6 min walk test (6 MWT) and BMI (weight/height^2^). All of the tests have high reliability and validity and the procedures for administering SFT are standardized and described in detail [41, 42]. The CSRT was excluded due to safety reasons for our study population, as it requires flexion of the back. The 6 MWT will of practical reasons be performed walking around a 40 m course. In a study from Sciurba et al. [43] there was reported no significant difference between walking courses of 15 to 50 m.

#### Health-related quality of life

HRQOL will be measured by the Quality of Life Questionnaire of the European Foundation for Osteoporosis (QUALEFFO-41) which is a disease-specific self-reported quality of life questionnaire containing 41 questions or items in five sections; pain, physical function, social function, perception of general health and mental function. These five sections can be evaluated separately or represented in a total score of all the 41 items [7]. Futhermore HR-QoL will also be measured by the Short Form 36 (SF-36). The SF-36 is divided into eight sub-scales (physical function, role limitations-physical, bodily pain, general health, vitality, social function, role limitations-emotional and mental health). The instrument is scored to a 0–100 scale for each sub-scale, the higher the score, the better the health status [44]. The SF-36 is shown to have high reliability and validity among older people [45].

#### Fear of falling

The Norwegian version of the Falls-Efficacy Scale international (FES-I) [46, 47] will be used to examine fear of falling. The FES-I assesses level of concern about falling on a 4 point scale during 16 activities of daily living. Scores range from 16 to 64 with higher scores indicating greater concern about falling.

#### Physical activity

To measure level of physical activity we will use the International Physical Activity Questionnaire Short Form (IPAQ –SF). IPAQ-SF measures self reported physical activity over the previous 7 days. It records 4 levels of physical activity; vigorous- intensity, moderate intensity, walking and sitting. The IPAQ-SF is frequently used as a measure of physical activity and is reported to have high reliability [48], its validity against objective measures of physical activity is questioned by Lee et al. [49].

### Adherence

Adherence will be calculated as the percentage completed of the total intervention days prescribed (24 sessions = 100%).

### Sample size estimation

The sample size is based on a substantial meaningful change in 10 m habitual walking speed [50]. Perera [50] defined the minimal detectable change as 0.05 m/s. A substantial meaningful change is defined as 0.10 m/s and the expected standard deviation in habitual walking speed is assumed to be 0.2 m/s based on findings by Perera [50]. This estimate requires 128 patients, 64 in each group, to obtain 80% statistical power with 5% significance level for an independent samples t-test. We will aim to include 150 in order to compensate for an estimate of 20% drop outs.

### Statistical procedure

The results of the study will be reported in accordance with CONSORT [51]. Statistical analysis is performed using statistical software as e.g. SPSS, Stata or R. The level of significance is 5%. Descriptive data will be reported as mean (standard deviation), median (range) or count (percent) as appropriate. The data will be analysed following the intention-to-treat principle. Between group differences in primary and secondary outcomes at 3 months follow-up will be assessed using linear regression models with respective outcome at baseline as covariate and the randomised group as factor (dummy variable), i.e. an ANCOVA analysis [52]. In addition, we will perform linear mixed model analysis for repeated measurements. We expect few or none missing data in baseline characteristics. However, due to possible drop-outs or participants unable to complete the outcome measurements, there might be missing data for both primary and secondary outcomes during follow-up. Linear mixed model for repeated measurement is able to statistically adjust for missing data associated with variables in the model, i.e. missing at random (MAR) structure. Further, we will also assess multiple imputation methods and sensitivity analysis for missing data in longitudinal studies. All tests will be two-sided. A statistical analysis plan will be performed in advance of each paper from the study.

## Discussion

The main purpose of the study is to evaluate the impact of a resistance and balance programme informed by expert panel recommendations for older women with osteoporosis and a history of vertebral fracture. We anticipate that the intervention described will have a positive impact on the primary outcome, 10 m habitual walking speed, as well as secondary outcomes such as HRQOL [3, 18, 23, 24]. Our study has a range of secondary outcomes which can give a broad picture of the women’s health and life situation. The presence of one or more osteoporotic vertebral fractures has a large impact on HRQOL [10], and findings from a 7 year follow up study shows that lowered scores on pain and disability does not fade away unless effective treatment is given [9]. QUALEFFO-41 and SF-36 are outcome measures applied to examine HRQOL and can add valuable knowledge whether exercise can improve HRQOL.

We rely on a systematic approach which corresponds with the guidance on developing and evaluating complex interventions [53]. Several issues related to the quality of the study have to be discussed, such as internal and external validity. Examples of methodological issues that may influence internal validity in a RCT include random allocation, blinding, outcome measures, sample size, drop-outs, statistical methods, and the participants’ adherence to the intervention. In the present study we are particularly concerned with the outcome measures and the participants’ adherence to the programme. Adherence to the programme will be of importance for the internal validity of the study. For some women it can be a challenge to be able to attend to the course twice a week for 12 weeks. In particular this can be challenging for the frailest of the participants. Motivational factors can also affect the adherence. Participants who don’t attend will be followed up with phone calls to motivate and find solutions so they can complete the programme as intended.

Implementation of research into clinical practice is of importance for the overall quality in health research [53]. If the intervention of this present study appears effective, one of its advantages is its applicability in the health care system. The programme requires little equipment, has low material costs and can easily be implemented in community based facilities. The women can perform many of the exercises at home without too much space and equipment to maintain strength and balance after the completion of the programme. The exercise programme can be performed in groups and is therefore more cost effective than one-on-one supervision. The design of group-based stationary circuit session allows the maintenance of the need for individual tailoring. Furthermore, the programme can also be used as a home-based exercise programme under supervision of health care professionals for those who are too fragile to attend to a community based group. Last but not least the exercise programme can also be used as home based“self-training” exercise based on welfare technology/telerehabilitation. The intervention will have potential to promote evidence-based decision-making and empower women with osteoporosis and vertebral fracture to remain in charge of their own lives.

### Limitations

Some limitations of this study should be noted. First, this study evaluates the programme in an urban area, which may limit its generalizability to rural parts and other states with varying demographic characteristics of older adult populations. Second, in this study we don’t recruit people with low walking speed in particular, and it therefore be less likely that we see an effect on the primary outcome. Furthermore, the Hawthorne effect should be considered [54]. Exercise intervention studies appeal to healthier and better-motivated individuals [55]. In this study the intervention is based on an exercise intervention of 12 weeks, twice a week. Frail women with osteoporosis and a history of vertebral fracture in particular may not be able to attend community-based classes twice a week, even though they might benefit even more than healthy women from an exercise intervention. Another limitation is the inclusion criteria, which restrict our findings to individuals living on their own and being free from severe cognitive symptoms. Again the frailest may not be included. General conclusions can only be drawn with caution and no conclusions about training results should be drawn beyond the female osteoporosis population with a history of vertebral fracture living at home. It is a risk of drop-outs and thereby missing data during follow-up. Impact of missing data will be assess with appropriate statistical methods as e.g. linear mixed models, multiple imputations and sensitivity analysis.

### Conclusion

There are a limited number of studies examining the effects of practical exercise programmes on health and quality of life in individuals with osteoporotic vertebral fractures. The proposed study will assess whether an evidence-based multicomponent exercise programme can enhance physical function as well as quality of life for women with osteoporosis and at least one vertebral fracture, as compared to no intervention. Further, the study will assess the effects of the exercise programme immediately post-exercise, and whether they are maintained after cessation. Findings from this study will contribute to the body of evidence available to clinicians to inform the management of people with osteoporotic vertebral fractures.

## Abbreviations

6MWT: 6 Minutes walk test
ADL: Activity of daily life
BMD: Bone mineral density
BMI: Body mass index
DXA: Dual X-ray absorptometry
FES-1: Falls-efficacy scale international
FR: Functional reach
FSST: Four square step test
HRQOL: Health related quality of life
IPAQ-SF: International Physical Activity Questionnaire Short Form
QUALEFFO-41: Quality of life questionnaire of the european foundation for osteoporosis
SF-36: Short form 36
SFT: Senior fitness test
